# The supportive care needs of parents with a child with a rare disease: results of an online survey

**DOI:** 10.1186/s12875-016-0488-x

**Published:** 2016-07-21

**Authors:** Lemuel J. Pelentsov, Andrea L. Fielder, Thomas A. Laws, Adrian J. Esterman

**Affiliations:** School of Nursing & Midwifery, Division of Health Sciences, University of South Australia, City East Campus, GPO Box 2471, Adelaide, SA 5001 Australia; Sansom Institute for Health Research and School of Nursing and Midwifery, University of South Australia, City East Campus, GPO Box 2471, Adelaide, SA 5001 Australia; School of Nursing and Midwifery, Faculty of Health, Keele University, Staffordshire, ST5 5BG UK; Australian Institute for Health and Tropical Medicine, James Cook University, Cairns, QLD Australia

**Keywords:** Parents, Rare diseases, Supportive care, Disease burden, Health providers, Needs assessment, Online survey

## Abstract

**Background:**

Parents caring for a child affected by a rare disease have unmet needs, the origins of which are complex and varied. Our aim was to determine the supportive care needs of parents caring for a child with a rare disease.

**Methods:**

An online survey was developed consisting of 45 questions (108 items) and separated into six domains. The survey included questions about perceived level of satisfaction with receiving care, experiences and needs of providing daily care, the impacts of disease on relationships, the emotional and psychological burdens of disease, and parents overall satisfaction with the support received.

**Results:**

Three-hundred and one parents from Australia and New Zealand completed the survey; 91 % (*n* = 275/301) were mothers, with 132 distinct rare diseases being reported. Fifty-four percent (*n* = 140/259) of parents were dissatisfied with health professionals’ level of knowledge and awareness of disease; 71 % (*n* = 130/183) of parents felt they received less support compared to other parents. Information regarding present (60 %, *n* = 146/240) and future services (72 %, *n* = 174/240) available for their child were considered important. Almost half of parents (45 %, *n* = 106/236) struggled financially, 38 % (*n* = 99/236) reduced their working hours and 34 % (*n* = 79/236) ceased paid employment. Forty-two percent (*n* = 99/223) of parents had no access to a disease specific support group, and 58 % (*n* = 134/230) stated that their number of friends had reduced since the birth of their child; 75 % (*n* = 173/230) had no contact with other parents with a child with a similar disease, and 46 % (*n* = 106/230) reported feeling socially isolated and desperately lonely. Most frequent emotions expressed by parents in the week prior to completing the survey were anxiety and fear (53 %, *n* = 119/223), anger and frustration (46 %, *n* = 103/223) and uncertainty (39 %, *n* = 88/223).

**Conclusion:**

This study is the first to develop an online survey specifically for use with parents to investigate their supportive care needs across a large and diverse group of rare diseases. The findings highlight that parents with a child with a rare disease have common unmet needs regardless of what disease their child has. Such information may allow health providers to improve child outcomes through improving parental supportive care.

## Background

Individuals and their families affected by rare diseases have traditionally received meagre attention from political, scientific and medical communities [1], with parents needs especially having received very little attention. For many parents caring for a child with a rare disease, they face a life-time of challenges and personal sacrifice, often with limited access to health services and support, and a lack of experienced health professionals to aid in their provision of care and decision-making for their child [2, 3].

The definition of a rare disease differs between countries, from 1:2000 individuals affected in Europe [4] to 1:10000 in Australia [5], and in the USA as one affecting fewer than 200,000 people [6]. Collectively, rare diseases are defined as a large diverse group of life-threatening or chronically debilitating diseases, the majority of which are genetic-based and originate in early childhood [7]. Rare diseases are further characterised by delayed or incorrect diagnosis, lack of scientific knowledge and information, a paucity of available care and treatment pathways, limited access to specialist support and services, and social and financial consequences [8–10]. There are approximately 6000–8000 rare diseases, many of which have no formal title and are difficult to diagnose [11]. As a group, rare diseases affect 6–10 % of the total population, or approximately 350 million people world-wide [8, 12], equating to 2.2 million Australians (or 1:19 people), including as many as 400,000 children [1].

In caring for their child with a rare disease, parents encounter daily challenges that are multidimensional, including changes in work patterns, income and domestic duties. They often require specialist health knowledge and additional health literacy, care giving skills and resources beyond those normally required by parents to effectively parent their child. Despite the fact that parents of children with chronic health problems encounter similar issues, parents of a child with a rare disease face many additional problems characteristic of rare diseases, such as: delayed or undetermined diagnosis, limited access to health information and resources, support groups (if existing) that are geographically scattered and them feeling socially isolated [13, 14].

Although recently there have been significant gains in biomedical research being conducted into rare diseases [6], very few social studies exist which focus on the supportive care needs of parents caring for a child with a rare disease. Studies that have, are typically restricted to a single disease/group of related diseases, involve small sample sizes and are limited to a specific country or culture [15]. Of the studies which have attempted to identify the supportive care needs of parents in a comprehensive fashion, these have related to paediatric cancers [16, 17]. An exception was an Australian-based pilot study [1] which surveyed 30 families of children diagnosed with genetic metabolic disorders to assess the impacts of disease on the family. That study highlighted that families are emotionally and financially stressed, they desire better communication and coordination of care, feel frustrated with the diagnostic process, value improved access to information, and require greater social and psychological support.

The lack of research recognising the burden of care on parents is surprising, given that the burden of caring for a child with a chronic disease typically falls on parents [18]. Further, there currently exists a paucity of studies which examine the full constellation of needs of parents regardless of what rare disease their child has. An exception is a recent scoping review by the authors [19] which sought to address this gap by providing a detailed synopsis of parental supportive care needs in rare diseases. As a developed country without a national strategy for rare diseases [20], Australia, in-particular, is in need of research investigating the impacts and supportive care needs of rare diseases in families. Australia would potentially benefit from having all-inclusive research to begin to coordinate a ‘whole-of-government’ approach to meeting the needs of families impacted by rare diseases [20].

It is envisaged that such research may lead to more appropriate individualised supportive care for parents and their families. It will inform health providers how to tailor support and implement services for families affected by rare diseases to improve family outcomes in the future.

### Aim

The aim of this research was to determine the supportive care needs of parents caring for a child with a rare disease.

## Methods

### Target population

The target population were any mothers and fathers with a child (18 years or younger) with a rare disease, or whose child was suspected of having a rare disease but was not yet formally diagnosed. Parents of a child with an unconfirmed disease were included as many parents have a child suffering from a rare disease with no formal diagnosis [21]. We asked parents to state what rare disease their child was diagnosed with, and these were cross-checked against the Orphanet database of rare diseases [22]. Results reported are from parents living in Australia (AUS) and New Zealand (NZ).

### Development of the survey

The survey was created, based on a scoping review [19] and a focus group study [23] with parents investigating the supportive care needs of parents with a child with a rare disease. The survey was constructed using the web-based survey development software SurveyMonkey® (www.surveymonkey.net). The final survey consisted of 45 questions (108 items) and was separated into six main domains; 1) Demographics; 2) Equity in care; 3) Practical care needs; 4) About your relationships; 5) About your Emotions; and 6) Summary. The purpose of the survey was to identify the supportive care needs of parents across each domain at the time of completion of the survey.

The first domain “Demographics” (13 items) collected information including; number of affected children and type of rare disease (if known), and whether the respondents themselves were affected/carriers of the disease. The second domain, “Equity in Care” (11 items) pertains to respondents perceived level of overall satisfaction with the care that they have received from government and non-government services (e.g., parent support groups), as well as from family and friends. Respondents were also asked to report whether they thought the care that they received was the same, less or more than parents with a chronically ill child. The third domain, “Practical care needs” (43 items), relates to the experiences and needs of respondents in providing daily care to their child with a rare disease. Respondents were asked to rate their level of need across a number of areas including; information and communication, access to supports and current financial situation. The fourth domain, “About your relationships” (12 items) asks respondents to rate the impacts of their child’s disease on their relationships including partner, siblings and others. Domain five, “About your Emotions” (24 items), sought to identify existing emotional and psychological needs of respondents. Questions were targeted at how well respondents and their partner/spouse were coping emotionally, and whether they/their partner had developed any psychological health problems since the birth of their child with a rare disease. The final domain, “Summary” (2 items) asked respondents to rate their overall satisfaction with the support they receive for their child and to suggest any other supportive care needs not covered in the survey. In addition to closed questions, mainly in the form of multi-item 5-point Likert scales, there were six open-ended free text response items imbedded across the six domains to illicit any new information that might not have been identified in the scoping review or focus groups. Respondents were encouraged to complete these open-ended questions.

### Source of individual questions

While the survey was constructed based on the previously reported scoping review and focus group research, some of the individual items were founded on existing validated tools, namely the Dyadic Adjustment Scale (DAS) and the Family Needs Assessment Tool (FNAT), and adapted to suit the survey. The DAS is a validated instrument that has been frequently used for measuring satisfaction in relationships [24]. It was considered appropriate for this survey as it has been previously used to provide a general measure of the overall quality of relationships [25]. A single item from the DAS was modified for use in the survey for one item relating to partner relationships in domain four. The FNAT was designed for assessing family coping/functioning and level of social support in caring for a chronically ill child [26]. This validated self-reported tool helps identify families’ needs, as perceived by the parents themselves rather than by health professionals, and relates to information, services and access to health care. Fourteen items of the FNAT were modified for inclusion in the survey relating to the practical care needs (domain 3) of parents in caring for their child with a rare disease.

### Reliability, validity and pilot testing of the survey

Aside from the demographic questions which asked factual information (e.g., how many of your children are affected by a rare disease?), the majority of items within the survey are attitudinal/opinion-based questions. These items were therefore formally assessed for validity and reliability, and pilot tested.

### Validity

A draft of the survey was reviewed by three content experts to assess it for content and face validity. These individuals were considered content experts as they were either working in the area of rare diseases or had survey development expertise. Content experts were asked to consider whether the questions appeared to have face validity, whether they felt questions should be modified/removed, and whether they felt additional questions should be added. Content experts were asked to rate each attitudinal item of the survey out of a score of 0 and 2 (0 = relevant and 2 = not relevant). Those that were rated as “not relevant” were removed or modified. This occurred with 12 items. Examples of the types of modifications made to the survey included: wording of question items to make them clearer, an ‘other/ N/A’ response option added to several Likert items and definitions given to explain terms within items.

### Reliability

Reliability of the survey was established using a test-retest analysis involving 12 parents who took part in the previously reported focus group study (Pelentsov, Fielder & Esterman, accepted). Parents were asked to complete the online survey, and a week later were asked to complete the same survey a second time. Intraclass Correlation Coefficient (ICC) scores ranged between 0.29 (95 % CI −0.28–0.71) and 0.93 (95 % CI 0.78–0.98). The majority of ICC scores (*n* = 53/71, 75 %) were above 0.6 ICC. Seven items scored below 0.4 ICC, which were individually reviewed. A decision was made to either; remove those low scoring items altogether, modify the wording to make it clearer or ignore the ICC value with a justified reason (e.g., a single respondent reported significant differences in their responses between surveys).

### Pilot testing of survey

Pilot-testing of the survey was conducted with three parents who previously took part in the focus group study (Pelentsov, Fielder & Esterman, accepted), but were not involved in the reliability testing phase. The first author (LP) met face-to-face with each participant and moderated as they completed a paper-based copy of the survey. Any questions that each participant had regarding the content of the survey were answered/ addressed. Subsequent final changes to the survey were made following participant feedback and the “*Parental Needs Survey*” was in its final format.

### Dissemination of the *Parental Needs Survey*

A variety of approaches were used to disseminate the survey to the widest audience possible, including promotion through national and international peak bodies, paediatric hospitals and genetic departments, and community/support groups. Emails were sent to national and international peak bodies requesting their support to distribute the survey link to other organisations and member groups. The following peak bodies were instrumental in the dissemination of the survey; Rare Voices Australia (RVA), Genetic Alliance Australia (AGSA), Genetic and Rare Disease Network (GaRD), Genetic Support Network of Victoria (GSNV) and the New Zealand Organisation for Rare Disorders (NZORD). Emails were also sent to specific rare disease support/community groups detailing the research and requesting their support to forward the survey link to their members to complete. The survey was online and available for completion over a 4-month period from February-May 2015, following which the survey was closed.

### Ethical considerations

Ethics approval was granted by the University of South Australia Human Research Ethics committee (protocol: 0000031772). Informed consent was obtained when participants completed the survey online. The opening page of the survey presented participants with the overall purpose of the study as well as acknowledging that some of the questions may be considered by respondents to be of a sensitive nature. Anonymity was safeguarded as there was no personal identifying details obtained from respondents who completed the online survey. The final page of the survey asked participants to email the primary author (LP) if they wanted to be informed of the results of the survey.

### Statistical analysis

Since epidemiological data for most rare diseases is not available [27], there was no sampling frame to calculate desired response rates. Data were exported from Survey Monkey (www.surveymonkey.net) to SPSS version 22 (SPSS, Inc., Chicago, Illinois). Initial descriptive analysis included counts and percentages for categorical variables, and means and standard deviations for continuous variables.

Summative scores were calculated for the following twelve domains: health professionals, information, education, confidence, financial, access supports, partner, siblings, friends, other parents, isolation and emotions. We wished to determine which of the created domain scores were best jointly associated with overall dissatisfaction with support received using regression modelling. The question on overall satisfaction was asked on a five-point Likert scale; however, since the responses to this question were highly skewed, we dichotomised the responses into “Very dissatisfied” and “Dissatisfied” versus all other responses, and used logistic regression for the analysis. For each domain, the patterns of odds ratios (OR) for each item was similar and therefore, summative scores were calculated. A stepwise approach was taken, with the Bayesian Information Criterion (BIC) used to assess each model. The model with the lowest BIC score was selected as the final model.

## Results

### Domain one: Demographic details

A summary of all parent characteristics are provided in Table 1. Three hundred and one parents responded to the survey, the majority were mothers. Most respondents had at least one child living with them with a rare disease. A total of 132 distinct rare diseases were reported by parents in this survey. Twelve parents reported that their child remained undiagnosed or was yet to receive a confirmed diagnosis. One third of parents who completed the survey were living in a country/rural area.

**Table 1:** Demographic details of parents with a child with a rare disease

Parent characteristics	n	%
Gender		
Mother	275	91.4
Father	26	8.6
Total	301	100.0
Number of children with a rare disease		
1	259	86.0
2	36	12.0
3 or more	6	2.0
Total	301	100.0
Parent status of disease		
Diagnosed as a carrier/ full disease	85	28.2
Suspected carrier/ full disease	21	7.0
No carrier/disease	195	64.8
Total	301	100.0
Age		
15–24	8	2.7
25–34	84	27.9
35–44	131	43.5
45–54	66	21.9
55+	12	4.0
Total	301	100.0
Country and region of residence		
Australia (total)	285	100.0
Metropolitan/ City	190	66.7
Rural/ Country area	95	33.3
New Zealand (total)	16	100.0
Metropolitan/ City	10	62.5
Rural/ Country area	6	37.5
Total	301	100.0
Marital status		
Single/ Never married	5	1.7
Married/ Defacto	258	85.7
Widowed	7	2.3
Separated/ Divorced	31	10.3
Total	301	100.0
Respondent’s employment status		
Of parent		
Full-time wage earner	52	18.5
Part-time wage earner	101	36.1
Non-wage earner	127	45.4
Total	274	100.0
Partner’s employment status		
Full-time wage earner	184	79.6
Part-time wage earner	24	10.4
Non-wage earner	23	10.0
Total	231	100.0

Table 2 presents results from across domains two through five, and shows where parents identified the greatest needs.

**Table 2:** Parental reported needs across aspects of caring for a child with a rare disease

Variables	n	(%)	95 % CI
Parents receiving little or no support from (*n* = 262)			
Government services (i.e., public hospitals)	97	(37.0)	30.0–45.2
Non-governmental agencies (i.e., support groups)	88	(33.6)	26.9–41.4
Family and friends	78	(29.8)	23.5–37.2
Parents dissatisfied or extremely dissatisfied with support from health professionals			
Having a consistent team of health professionals taking overall responsibility for your child’s health (*n* = 251)	65	(25.9)	20.0–33.0
The overall support that you get from health professionals for your child (*n* = 257)	61	(23.7)	18.2–30.5
Feeling that you are part of a health care team looking after your child (*n* = 259)	72	(27.8)	21.8–35.0
How much health professionals know about your child's disease (*n* = 259)	140	(54.1)	45.5–63.8
Gaining a formal diagnosis for your child (*n* = 248)	73	(29.4)	23.1–37.0
Help with family planning (*n* = 173)	81	(46.8)	37.2–58.2
Parents in desperate need for information (*n* = 240)			
Information about my child’s disease	102	(42.5)	34.7–51.6
Information on how my child will grow and develop	134	(55.8)	46.8–66.1
Information on how to manage my child’s behaviour	85	(35.4)	28.3–43.8
Information about services that are presently available for my child	146	(60.8)	51.4–71.5
Information about services my child might receive in the future	174	(72.5)	62.1–84.1
Parents in desperate need of support with			
The educational needs of my child (*n* = 231)	80	(33.3)	27.5–43.1
Teaching my child about their disease (*n* = 218)	49	(20.4)	16.6–29.7
Explaining my child’s disease to his or her siblings (*n* = 224)	39	(16.3)	12.4–23.8
Explaining my child’s disease to my parents or relatives (*n* = 237)	33	(13.8)	9.6–19.6
Responding when friends, neighbours, or others ask questions about my child (*n* = 237)	39	(16.2)	11.7-22.5
Explaining my child’s disease to other children (*n* = 233)	57	(23.8)	18.5-31.7
Explaining my child's disease to his/her educator and school (*n* = 233)	58	(24.2)	18.9–32.2
Communicating with healthcare professionals (*n* = 238)	40	(16.7)	12.0–22.9
Parents in desperate need for access to respite services (*n* = 239)			
Babysitters capable of caring for your child	81	(33.9)	26.9–42.1
Formal respite care providers capable of caring for your child	84	(35.1)	28.0–43.5
Day care program or preschool for your child	49	(20.5)	15.2–27.1
School that is able to care for your child	85	(35.6)	28.4–44.0
Parents in need of professional/expert support (*n* = 223)			
Marriage counsellor	44	(19.7)	14.3–26.5
Psychological counsellor	106	(47.5)	38.9–57.5
Financial advisor	71	(31.8)	24.9–40.2
Social worker	72	(32.3)	25.3–40.7
Genetic counsellor (for family planning advice)	107	(34.5)	39.3–58.0

All items are 5-point Likert scales dichotomised to the two categories of greatest need

### Domain two: Equity in care

Table 2 shows the percentage of parents who reported receiving little or no support from government agencies, non-government services, or family or friends. Although the types of support available from these varied sources likely takes very different forms, notably, 7 % of all parents (*n* = 18/262; 95 % CI 4.1–10.9 %) reported having received no support at all from any of these. In open-ended responses by parents, reasons include: child’s disease not eligible/did not qualify for government support and no follow-up/ongoing provision of support; no available parental support groups/ contact with other parents; family do not live nearby/unable to provide support or simply were not interested/willing to help. The open-ended response from one parent summed up the views of many:

“I don’t receive any support from government. I pay for all his therapies privately. There is no support groups around. And I have never met anyone else with the same condition as my son. Majority of family are just not interested in assisting or even getting to know my son” (Mother, child with Phelan-McDermid syndrome).

Respondents were dissatisfied with various aspects of the provision of care for their child by health professionals. Parents were most dissatisfied by the level of knowledge of health professionals regarding their child’s disease and with receiving help with family planning.

When asked whether they felt they received more, less or the same support for their child compared to other parents with a chronically sick child, 71 % (*n* = 130/183; 95 % CI 59.4–84.4 %) of parents felt that they received less support.

### Domain three: Practical care needs

**Fig. 1:**
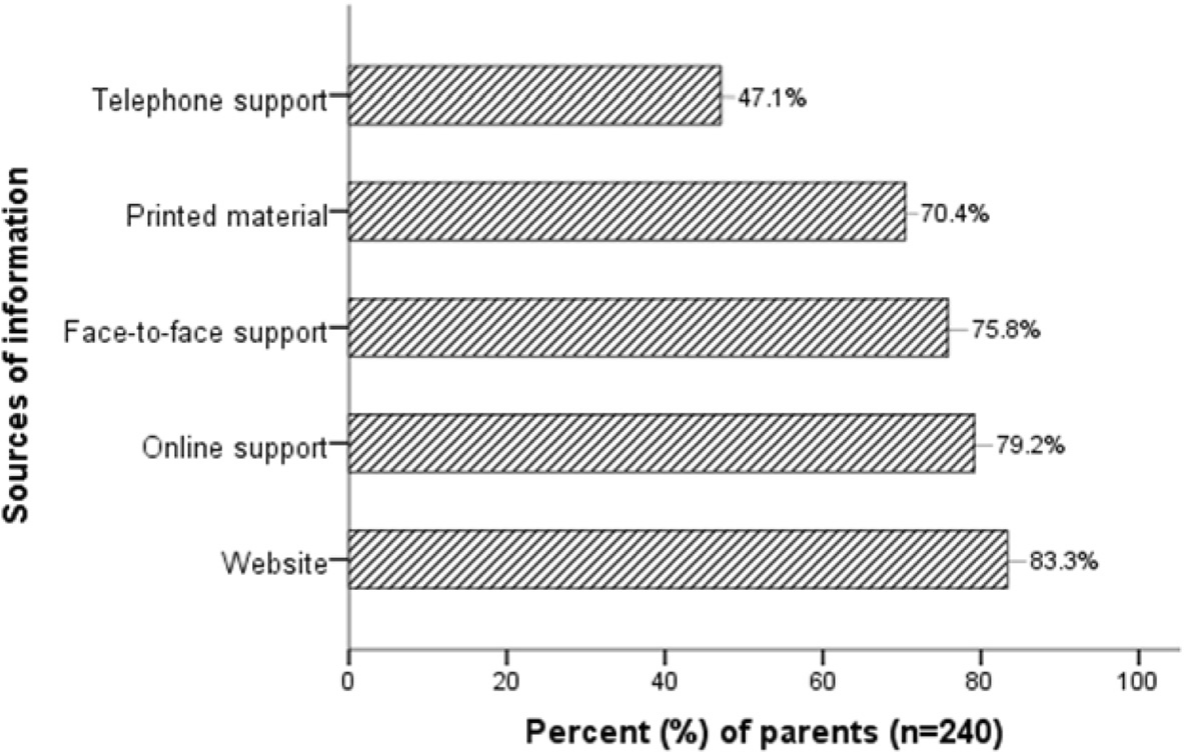
Sources of information considered most helpful to parents

Of the information available to them, parents felt the information was both helpful (70 %, *n* = 167/240; 95 % CI 59.4–81.0 %) and easy to understand (75 %, *n* = 179/240; 95 % CI 64.1–86.4 %). Yet, parents still reported needing more information. In particular, parents were most in need of information regarding services that were presently available for their child and services that their child might receive in the future (Table 2). Parents found two sources of information to be of most help to them, namely websites (83 %, *n* = 200/240; 95 % CI 72.2–95.7 %) and online support (79 %, *n* = 190/240; 95 % CI 68.3–91.3 %) (Fig. 1).

The educational needs of their child appeared to be of highest concern to parents, with a third reporting they needed help with their child’s educational needs, followed by help with explaining their child’s disease to his/her educator and school and then to other children (Table 2).

In terms of managing all aspects of daily care for their child with a rare disease, most parents said they felt confident in managing these daily tasks. Aspects of care parents felt confident in handling included: ability to assess and care for their child’s health needs (85 %, *n* = 204/239; 95 % CI 74.0–97.9 %), respond to emergency situations (84 %, *n* = 201/239; 95 % CI 72.9–96.6 %), discuss any health concerns related to their child’s disease with health professionals (89 %, *n* = 213/239; 95 % CI 77.6–100.0 %) and make decisions about the health of their child (94 %, *n* = 225/239; 95 % CI 82.2–100.0 %).

**Fig. 2:**
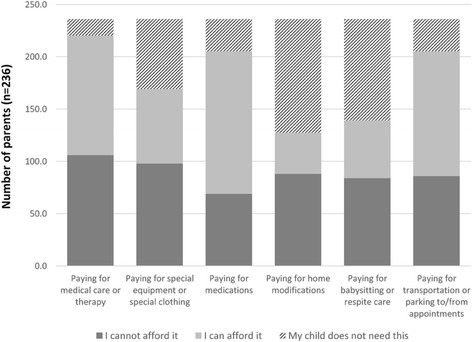
Affordability of different needed services at the time of the survey

Almost half of parents (45 %, *n* = 106/236; 95 % CI 36.8–54.3 %) said overall, they were not coping financially with the costs associated with caring for their child with a rare disease. Figure 2 shows the level of affordability of different services required by the parents at the time of the survey. Paying for medical care and therapy was the aspect that most parents could not afford.

The majority of parents said that their child’s needs had impacted on their work/employment status; with 38 % (*n* = 90/236; 95 % CI 30.7–46.9 %) of parents having to either reduce their working hours or quit working altogether (34 %, *n* = 79/236; 95 % CI 26.5–41.7 %). For many of the parents, partners’ employment status was also affected with 25 % (*n* = 53/211; 95 % CI 18.8–32.9 %) having to reduce their working hours, and 29 % (*n* = 61/211; 95 % CI 22.1–37.1 %) needing to work longer hours in order to cope financially.

Many of the parents in this study indicated difficulties with accessing certain supports with 42 % (*n* = 99/233; 95 % CI 34.5–51.7 %) of parents reporting not having access to a parental support group specific to their child’s disease, and 37 % (*n* = 86/233; 95 % CI 29.5–45.6 %) saying they did not have access to a health professional who understood their child’s complex health needs. Almost half of parents (48 %, *n* = 111/233; 95 % CI 39.2–57.4 %) said they had no access to counselling supports (e.g., psychologist, social worker, Psychiatrist).

### Domain four: About your relationships

The majority of respondents (73 %, *n* = 149/203; 95 % CI 62.1–86.2 %) said having a child with a rare disease has impacted on the relationship with their partner. Open-ended comments from parents suggest that the impacts on the relationship are both positive:

“I think it has brought us closer together. We are going through something together that no one else really understands. We have had to rely upon one another and have learnt to appreciate each other more. We are stressed, tired and worried a lot of the time, but generally we are good supports for each other.” (Mother, child with 49 XXXXY syndrome)

And negative:

“We sail like ships in the night. I am dealing with the caring and house so that my partner can work harder for the business to keep us financially afloat. We are utterly exhausted and we don't have money for holidays and have no time alone together.” (Mother, child with Chronic Intestinal Failure)

Many parents (54 %, *n* = 99/185; 95 % CI 43.5–65.2 %) felt that they did not give the child’s other siblings enough of their time or attention. For example:

“It has strained us by not being able to give a normal life to our healthier children. It separates us into two families essentially.” (Mother, 2 children with mitochondrial encephalomyopathy, lactic acidosis and stroke-like episodes – MELAS)

Parents in the survey also reported having a child with a rare disease has impacted on their friendships, with more than half of parents (58 %, *n* = 134/230; 95 % CI 48.8–69.0 %) stating that their number of friends had reduced since the birth of their child with a rare disease. The majority of parents (75 %, *n* = 173/230; 95 % CI 64.4–87.3 %) said that they have not come into contact with other parents with a child with a similar problem to them. As a result, almost half of parents (46 %, *n* = 106/230; 95 % CI 37.7–55.7 %) reported feeling socially isolated. Further, when asked how lonely they were feeling in the past month, 46 % (*n* = 105/230; 95 % CI 37.3–55.3 %) of parents said that they felt desperately lonely.

### Domain five: About your emotions

**Fig. 3:**
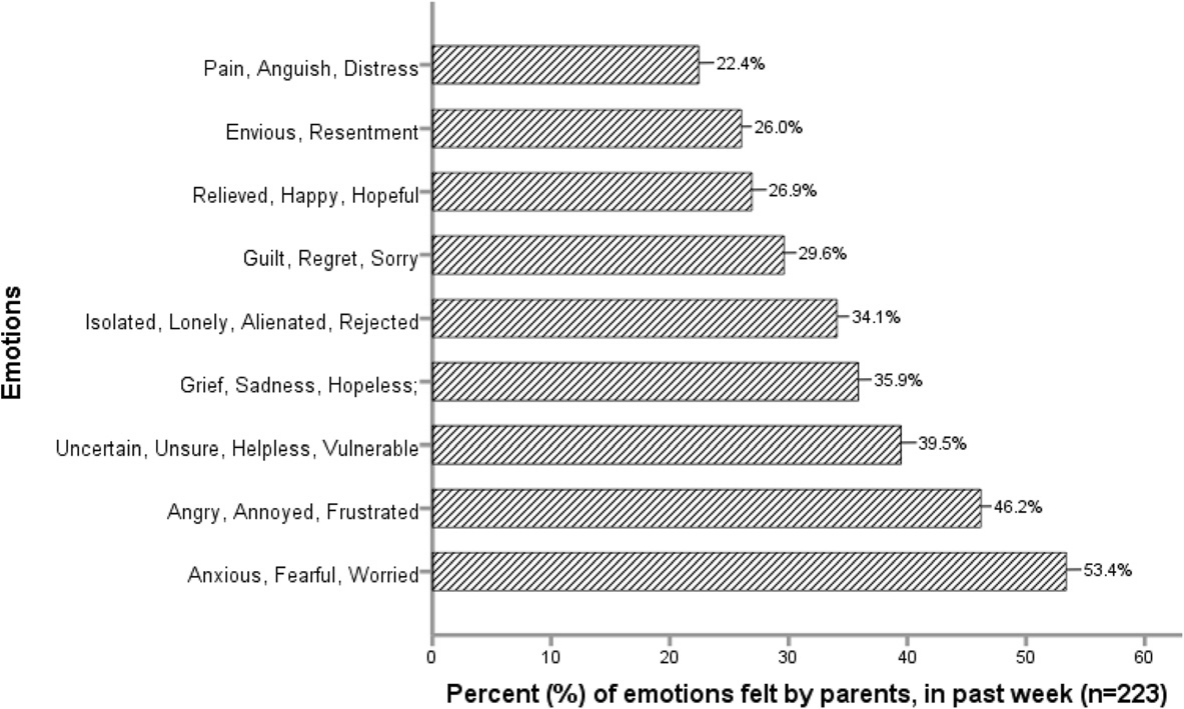
Types of emotions felt by parents in the week prior to completing the survey

Figure 3 shows the types of emotions most frequently expressed by parents in the week preceding the survey. Parents expressed feelings of; anxiety, fear and worry, followed by anger, annoyance and frustration, and uncertainty, helplessness and vulnerability.

Twenty percent (*n* = 44/219; 95 % CI 14.6–27.0 %) of parents reported taking medication to help them cope emotionally in the week prior to completing the survey. A number of the parents indicated they were being treated for mental health problems since the birth of their child with a rare disease; 37 % (*n* = 83/223; 95 % CI 29.7–46.1 %) of parents said they were being treated for depression, 41 % (*n* = 91/223; 95 % CI 32.9–50.1 %) for anxiety and 10 % (*n* = 23/223; 95 % CI 6.5–15.5 %) for other psychological problems. As one parent shared:

“I had a hard time coming to terms with her condition which put a strain on our marriage. Since her birth, I have suffered depression and have had episodes of extreme anxiety.” (Mother, child with Stickler syndrome)

With relation to partners, 22 % (*n* = 49/223; 95 % CI 16.3–29.0 %) were diagnosed and being treated for depression and 17 % (*n* = 37/223; 95 % CI 11.7–22.9 %) for anxiety and 5 % (*n* = 11/223; 95 % CI 2.5–8.8 %) for other psychological problems.

Table 2 shows the services that parents in the survey felt they needed most with almost half reporting they needed a psychological counsellor followed by a genetics counsellor/family planning expert.

### Domain six: Summary

The final item in the survey asked parents to rate their overall satisfaction with the support that they had received from any source for their child with a rare disease. Almost half of parents (45 %, *n* = 101/222; 95 % CI 37.1–56.3 %) reported they were dissatisfied with the overall level of support that they have received.

Table 3 shows the most important variables for parents’ overall dissatisfaction with care received. The final model contained four domains that were best jointly associated with parental dissatisfaction with support, namely: health professionals (i.e., their low level of knowledge and support provided), confidence (i.e., parents’ lack of confidence in caring for their child), financial (i.e., level of financial assistance needed), and emotional (i.e., level of emotional need of parents).

**Table 3:** Results of logistic regression of overall dissatisfaction with support received on domains of support

	Univariate analysis ^a^			Multivariable analysis ^b^		
Variable	Odds Ratio O.R.	95 % CI O.R.	Sig.	Odds Ratio O.R.	95 % CI O.R.	Sig.
Health professionals	0.770	0.704–0.842	<0.001	0.756	0.630–0.907	0.003
Information	0.814	0.761–0.871	<0.001			
Education	0.911	0.875–0.947	<0.001			
Confidence	0.859	0.799–0.922	<0.001	0.773	0.612–0.978	0.032
Financial	0.846	0.773–0.926	<0.001	0.847	0.729–0.984	0.030
Access supports	0.674	0.592–0.766	<0.001			
Partner	1.015	0.945–1.090	0.690			
Emotions	1.072	1.028–1.117	0.001	1.183	1.035–1.353	0.014
Siblings	0.644	0.503–0.825	<0.001			
Friends	0.535	0.413–0.692	<0.001			
Other parents	0.702	0.544–0.906	0.007			
Isolation	0.542	0.426–0.689	<0.001			

^a^ From binary logistic regression

^b^ Model selection based on minimising Bayesian Information Criterion (BIC)

## Discussion

This study is the first study to investigate the supportive care needs of parents caring for a child with any rare disease. A comprehensive survey was developed from the literature [19] and qualitative focus group data (Pelentsov, Fielder & Esterman, accepted) tailored specifically to parents caring for a child with a rare disease. The online survey targeted a large and diverse number of rare diseases and was demonstrated to be both acceptable and relevant to investigating parent’s supportive care needs. Responses to the *Parental Needs Survey* were received from parents of more than 130 distinct rare diseases across a number of important domains. These domains were; equity in care, practical care needs, relationships and emotional needs of parents. The findings from this study highlight that parents living with a child with a rare disease have unmet needs that are common regardless of what disease their child has. Such information is needed to inform health providers how best to tailor support and implement services for families affected by rare diseases with the eventual goal to improve future family outcomes.

### Health professionals

The lack of knowledge and provision of care and support by health professionals was identified as a supportive care need by parents in this study. Parents reported being dissatisfied with health professionals regarding; overall support received, level of knowledge and awareness of their child’s disease, gaining a formal diagnosis, help with family planning and feeling a part of the health care team looking after their child. More than half (54 %) of parents felt that health professionals lacked the necessary knowledge and awareness to properly care for their child with a rare disease. Parents also indicated that they desired having a consistent team of health professionals overseeing their child’s health care needs and for them to feel a part of that health care team. Dissatisfaction by parents towards health professionals due to a lack of knowledge and awareness of disease, poor communication and want of information has been previously reported, and is a common problem amongst parents caring for a child with a rare disease [19, 28–30]. Parents want to feel as if they belong to the health care team, to feel reassured that there is somebody taking ultimate responsibility for their child’s care and that their child’s health care needs are being met. Instead, parents often feel like they are a little bit of everyone’s problem, but no one’s actual responsibility (Pelentsov, Fielder & Esterman, accepted). These findings are consistent with previous studies that discuss parents’ feelings of frustration and concern on the part of health professionals’ lack of knowledge and understanding of their child’s rare disease, and how this negatively impacts on their quality and access to care [19, 31, 32]. Poor communication and limited information hinders parent’s ability to cope with their child’s care demands and managing their child’s health especially when crises arise [33–35]. Our study has shown that the poor quality of care received from health professionals is strongly associated with their overall dissatisfaction with the support that they receive for their child. Having a positive experience with health professionals has been shown to have a major, lasting influence on parents’ ability to cope and adapt to their child’s disability [28]. In addition to having good communication, health professionals across disciplines need to have a greater awareness and level of knowledge of rare diseases and the impact of these diseases on the individual and their family. Currently, rare genetic diseases and their impact do not feature strongly in health care curricula across disciplines. Therefore there is a need for greater integration of this information into training material [36]. Furthermore, specialist trained healthcare workers skilled in genetics and family counselling to provide parents of a child with a rare disease would be of great benefit.

### Social support

Many parents in this study clearly found the experience of caring for a child with a rare disease to be highly isolating. Social isolation, loneliness and feeling disconnected from mainstream society are common problems experienced by parents caring for a child with a rare disease, is one of the most stressful factors associated with caring responsibilities, and seen less often in parents caring for a child with a more prevalent chronic condition [19, 37]. The informal support provided by family and friends in particular, is a highly important source of social support and opportunity for interaction for parents, and in many cases, their primary coping system [35, 38]. Without access to this type of social support and respite, parents can become overburdened, emotionally exhausted and unable to strengthen resilience and cope daily with their burden of care [19]. An important finding from this study is that a significant number of the parents (58 %) reported having lost friends since the birth of their child with a rare disease leading to feelings of social isolation and loneliness. Parents find it difficult and/or lack the energy to maintain relationships and emotionally invest in them, often becoming withdrawn and limiting their interactions because of the everyday demands of their child’s disease [19]. As a consequence, maintaining social relationships and attending social gatherings becomes increasingly more difficult, and occurs less frequently. Conversely, seeing the struggle that parents face caring for their child with a rare disease can be confronting and unnerving for some [14]. Friends and family may struggle to fully comprehend the gravity of the parents’ situation and what they are going through, and as such, distance themselves from the parents [39]. Many parents (75 %) in this study stated that they had not come in contact with other parents who had a child with a similar condition to their child. Having peer support with other parents who share similar circumstances and receiving mutual support and encouragement is of central importance to parents. It provides parents with a shared social identity and sense of belonging, and enables parents to better cope with their situation, alleviate stress and feel empowered and supported to manage their child’s ongoing health care needs [40–42]. Due to the rarity of many of the rare diseases reported in this study, parents simply did not come in contact with other parents with a child with the same disease. For many rare diseases, parental support groups do not exist or are geographically scattered and difficult to access, and accessing online support groups can be equally as challenging for many rare diseases. There is a need for more generic online support groups for parents to access support regardless of what disease their child has, given that the current study has shown that parents share many similar supportive care needs irrespective of what disease their child has. Furthermore, websites and online support was considered the most beneficial source of information by parents in this study. Online parental support groups are relatively cost-effective and uncomplicated to implement as a meaningful intervention for supporting parents and families affected by rare diseases [43]. Therefore, developing a generic online support group that allows all parents access to meet other parents, exchange information, receive support, alleviate stress and develop a sense of belonging and community would greatly minimise the impact of isolation and loneliness in these parents.

### Confidence in caring for their child

Despite reporting a lack of support from health professionals, fortunately, the current study has shown that most parents reported feeling confident in looking after their child and their complex health needs. The majority of parents reported that they felt confident with regards to: assessing their child’s health needs, handling emergency situations when they arise, tailoring care, discussing their child’s disease with health professionals and making decisions. As a result of the reported lack of health professionals with knowledge of the disease, lack of resources and health literacy available to parents, many parents of a child with a rare disease feel they have no alternative but to assume the role of ‘expert’ regarding all aspects of their child’s health [32, 44, 45]. This includes, developing an expert knowledge base of the disease, sourcing information and understanding the disease journey, advocating with health professionals for treatment/interventions and being the one to make important decisions regarding their child’s ongoing care needs. However, the current study did highlight that there is a minority of parents who reported that they lacked the confidence and ability to properly care for their child with a rare disease. It is this minority group of parents who would most benefit from additional support. Parental insecurity and a lack of confidence in caring has been associated with a lack of information, social support and practical advice about the disease, leading to feelings of distress, anxiety and an inability to cope with the everyday demands of parenting a child with a rare disease [46]. In their dealing with parents, health professionals need to consider/assess parents’ perceived level of ability and self-confidence in caring for their child’s complex health needs, as well as their depth of knowledge and understanding of the disease and level of access to information and resources to assist them provide care and adequately parent their child, to gauge their required level of support.

### Parental well-being

Whilst a number of parents stated that they were diagnosed and being treated for depression (37 %) and anxiety (41 %) since the birth of their child with a rare disease, a causal link cannot be made between their care giver burden or perceived lack of support at the time of them completing the survey. However, there is considerable literature that implies a causal link between caregiver burden and associated depression and anxiety in parents of a child with a chronic illnesse [47, 48].

### Financial burden

Finally, financial distress was highly associated with parent dissatisfaction. Not only can treatments be expensive, depending on the condition, there might also be the need to purchase specialised clothes or equipment, undertake home modifications, and pay for constant visits to the hospital. These are compounded by the need for many parents to either cut down or stop work in order to care for their child. There have been surprisingly few studies that have explored the financial burden on parents of having a child with a chronic disease. However, Arafa et al. [49] found that the health related quality of life of parents caring for a child with heart problems was adversely impacted on by the parents’ financial situation. The majority of parents in the survey felt that they were under financial pressure. The difficulty is working out how these parents can be better financially supported. Unfortunately, many parents, especially those with a child not yet diagnosed, are not eligible for support services such as the Australian Nation Disability Insurance Scheme.

### National plan

The background section of this paper highlights that Australia is one of the few developed countries not to have a national strategy or plan for managing rare diseases. Notably, while these national plans exist in most developed countries, they focus on the needs of the affected children and fail to address the supportive care needs of parents. It is our belief that the results in this paper will be of help in assisting with the development of a national plan for Australia, especially with respect to services for affected families, and additionally, internationally, may encourage other countries to consider the supportive care needs of parents in their national strategies.

### Strengths and limitations

This present study is the first to investigate the supportive care needs of parents caring for a child with any rare disease using a valid and reliable online survey. In addition, a strength of the current study was the larger than expected sample size. Recruitment challenges and small sample sizes are common issues encountered by researchers undertaking work in the area of rare diseases [50]. However, this study recruited 301 parents of children affected by more than 130 distinct rare diseases, and is therefore likely representative of the rare diseases population. Despite this strength, the study includes limitations that should be taken into consideration. Firstly, there are a number of barriers related to using the Internet as a mode for survey distribution [51]. In particular, potential respondents may not have access to the internet/low internet coverage. Further, the survey was only distributed in English, meaning that there were likely parents who spoke a language other than English that were unable to respond, resulting in their views of parental supportive care needs not being included in this study. Secondly, of the total responses, the majority of respondents were mothers. Although repeated attempts were made to convey to parents the importance of investigating the supportive care needs of both fathers and mothers, fathers accounted for only a small number of survey responses (*n* = 26/301, 8.6 %) and thus, their perspectives on parental supportive care needs may remain under-represented. Having the perspectives of both the mother and father on the impacts that a rare disease has on the family would enable a richer description of the emotional, psychological and physical consequences, as well as, the everyday burdens experienced by parents caring for a child with a rare disease [52]. By targeting peak bodies for recruitment, there is clearly a possibility that respondents are more likely to belong to a support group, and thus take a greater interest in their child’s disease. Finally, anyone completing a survey, self-selects to do so, and there is always the possibility of selection bias. Due to the above limitations, it is unlikely that the results are generalizable to all parents of a child with a rare disease, in particular fathers and parents from non-English speaking countries.

## Conclusion

Our study is the first to develop an online survey specifically for use with parents with a child with a rare disease to investigate parental supportive care needs across a large and diverse group of rare diseases. Notably, this study highlights that parents caring for a child with a rare disease share common needs, irrespective of what disease their child has. This study gives health providers clearer direction on where to focus future efforts/attentions in order to improve delivery of care and access to support. In particular, health providers should be aware of the frustrations felt by parents due to a lack of knowledge and awareness of disease by health professionals, the impact that caring for a child affected by rare disease has on family relationships, that parents will likely be in an emotionally vulnerable state and suffering from financial distress. Based on the above findings, monitoring parents overall satisfaction with receiving care needs to be a priority, and can be improved by focusing on four main areas; health professional knowledge and understanding of disease and its impacts on families, ensuring parents feel confident in caring for their child and making decisions, providing improved financial support for families and monitoring their emotional wellbeing. There is a need for more research to be conducted into the supportive care needs of families affected by rare diseases, particularly the development of a tool for use by health professionals to identify and measure unmet supportive care needs of families, both at the individual and population level. This will potentially enable more tailored support, and improve access to health care and support services for the wider rare disease community.

## Abbreviations

AUS, Australia; BIC, Bayesian information criterion; DAS, dyadic adjustment scale; FNAT, family needs assessment tool; GA Aus, genetic alliance Australia; GaRD, genetic and rare disease network; GSNV, genetic support network of Victoria; NZORD, New Zealand organisation for rare disorders; OR, odds ratios; RVA, rare voices Australia
